# Shape Preserving Single Crystal to Amorphous to Single Crystal Polymorphic Transformation Is Possible

**DOI:** 10.1021/jacs.1c08590

**Published:** 2021-11-23

**Authors:** Olivier Renier, Guillaume Bousrez, Glib V. Baryshnikov, Veronica Paterlini, Volodymyr Smetana, Hans Ågren, Robin D. Rogers, Anja-Verena Mudring

**Affiliations:** † Department of Materials and Environmental Chemistry, Stockholm University, 10691 Stockholm, Sweden; ‡ Division of Theoretical Chemistry and Biology, School of Engineering Sciences in Chemistry, Biotechnology and Health, KTH Royal Institute of Technology, 10691 Stockholm, Sweden; § Department of Physics and Astronomy, Uppsala University, 75120 Uppsala, Sweden; ∥ College of Arts & Sciences, The University of Alabama, Tuscaloosa, Alabama 35487, United States; ⊥ Department of Chemistry and iNANO, Aarhus University, 8000 Aarhus C, Denmark; ○ Laboratory of Organic Electronics, Department of Science and Technology, Linköping University, SE-60174 Norrköping, Sweden

## Abstract

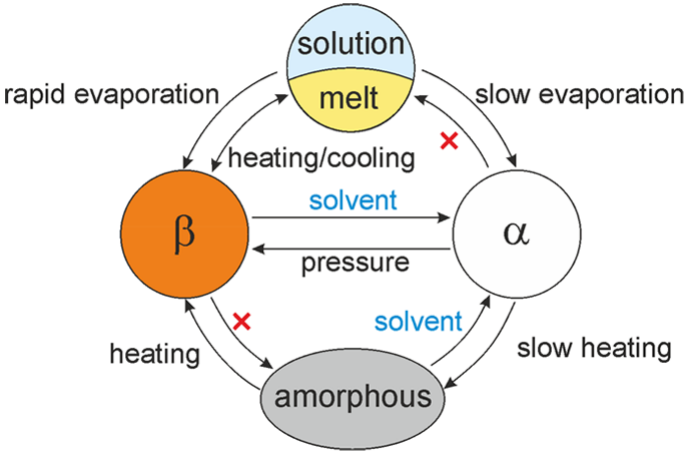

Many crystalline materials form polymorphs and undergo solid–solid transitions between different forms as a function of temperature or pressure. However, there is still a poor understanding of the mechanism of transformation. Conclusions about the transformation process are typically drawn by comparing the crystal structures before and after the conversion, but gaining detailed mechanistic knowledge is strongly impeded by the generally fast rate of these transitions. When the crystal morphology does not change, it is assumed that crystallinity is maintained throughout the process. Here we report transformation between polymorphs of ZnCl_2_(1,3-diethylimidazole-2-thione)_2_ which are sufficiently slow to allow unambiguous assignment of single crystal to single crystal transformation with shape preservation proceeding through an amorphous intermediate phase. This result fundamentally challenges the commonly accepted views of polymorphic phase transition mechanisms.

## Introduction

Polymorphism, the ability of a particular compound to exist in two or more crystalline states, is widespread in nature^1−4^ and represents significant scientific and commercial interest.^5−7^ Many polymorphic materials undergo solid–solid transitions^1−4^ between different forms as a function of temperature or pressure, and since a material’s chemical, physical, and biological properties depend on their crystalline form, control of these transformations is critical. Unfortunately, mechanistic understanding of the conversion pathways between polymorphs and the factors directing them is limited by the generally fast kinetics of such processes.^7−9^ Many transformations, particularly those of first order, seem to require a reconstruction of the crystal lattice, while in single crystal to single crystal (SC–SC) transitions both polymorphs typically exhibit close structural relationship,^10^ and it is assumed that crystallinity is maintained throughout the process.

Kinetics plays a significant role in phase transformations^11^ and frequently restricts our ability to closely monitor them. Only sufficiently slow processes can be followed closely with a set of conventional complementary techniques and investigated in detail. Herein, we present the shape preserving, polymorphic SC–SC phase transformation between polymorphs of ZnCl_2_(C_2_C_2_ImT)_2_ (C_2_C_2_ImT = 1,3-diethylimidazole-2-thione) which is slow enough to unambiguously determine an intermediate amorphous phase.

## Results and Discussion

**Figure 1:**
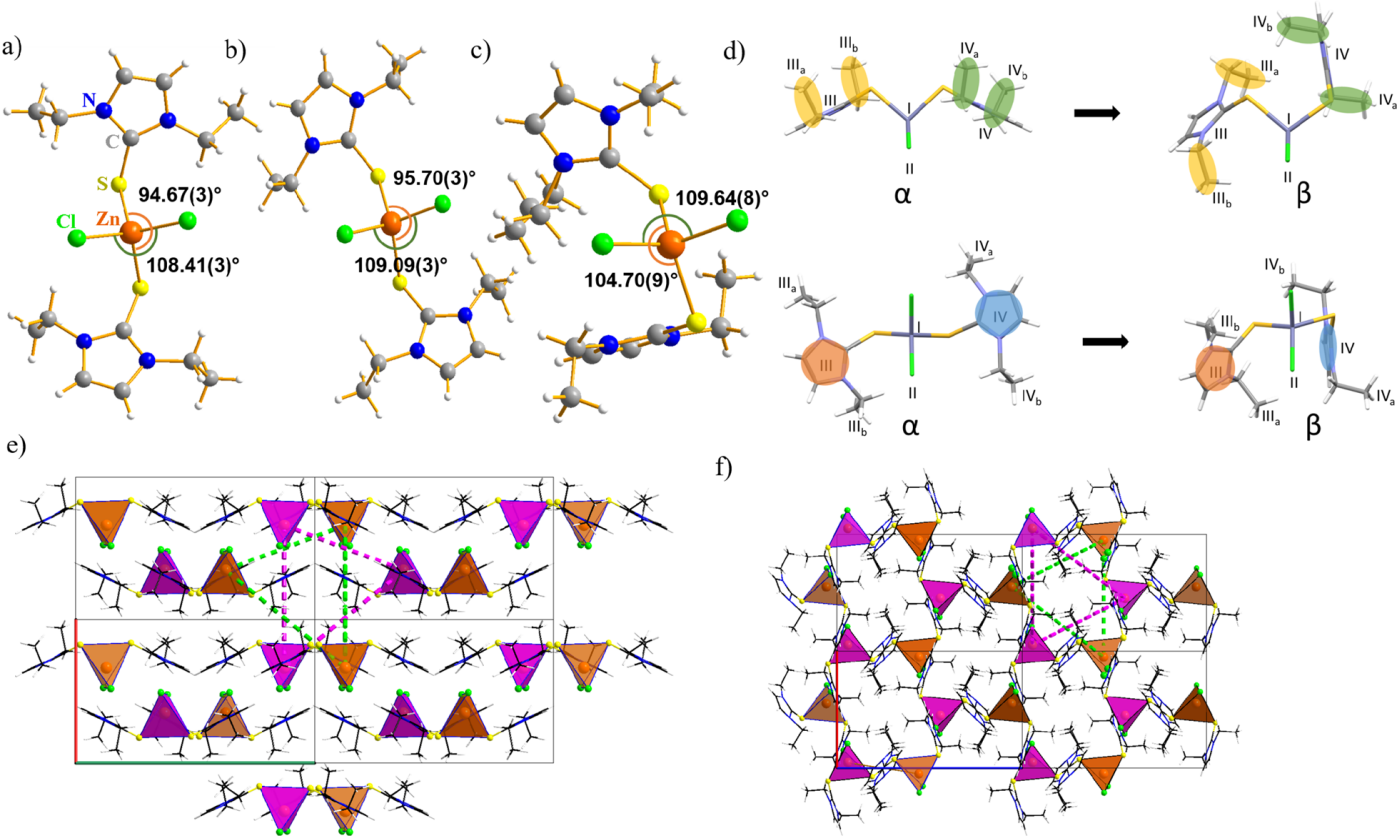
Molecular units for ZnCl_2_(C_2_C_2_imT)_2_ as observed in α (a, b) and β (c); illustration of the configurational and conformational changes between α and β (d); projections of the crystal structures of α (e) and β (f). ZnS_2_Cl_2_ tetrahedra in alternating layers are visualized in different colors. Crystallographic axes are color-coded: *a*, red; *b*, green; *c*, blue.

The reaction of ZnCl_2_ with 2 equiv of C_2_C_2_ImT in water followed by slow evaporation under ambient conditions resulted in large, mm-sized, colorless single crystals (see Figure S2 for details), confirmed by single-crystal X-ray diffraction analysis (SCXRD) to be ZnCl_2_(C_2_C_2_ImT)_2_, a typical coordination compound (Figure 1a,b) with Zn(II) in a (distorted) tetrahedral coordination environment of two Cl and two S atoms. During grinding of the crystals to a powder with a mortar and pestle to confirm the identity of the bulk via powder X-ray diffraction (PXRD), an immediate color change to orange was observed, and the PXRD diffractogram did not match the pattern calculated from SCXRD structure analysis. A similar change in color was noticed when attempting to cut larger single crystals with a scalpel. Here it was observed that while exerting mild mechanical impact, the color of the crystal changed but not its morphology although the crystalline specimen separated into multiple pieces.

Larger crystals of orange color could be obtained by rapid crystallization of ZnCl_2_(C_2_C_2_ImT)_2_ from aqueous solution under dynamic vacuum (rotary evaporator, 40 °C). SCXRD unambiguously revealed the orange phase (see also Figure S2), here designated as form β, to be a polymorph of the colorless form (referred to as form α). PXRD data confirmed quantitative conversion of α to β upon grinding (Figure S4).

Both polymorphs of ZnCl_2_(C_2_C_2_ImT)_2_ feature Zn(II) coordinated by two chloride anions and two thione ligands in, albeit differently, distorted tetrahedral coordination environments. The conformations of the thione ligands and the ethyl groups within the ligands (Figure 1c) display significant differences in their orientations. The ethyl chains in β adopt the thermodynamically more favorable *trans* form (cf. DFT calculations, *vide infra*). In addition, the mutual thione orientation within a complex in α can be considered flared out in a butterfly shape, while in β the thione ligands align almost orthogonal to one another (Figure 1d).

The structural relationship between α and β can be best described by a rotation of the imidazole rings around the Zn–S bonds and a rearrangement of the ethyl groups and the position of one of the imidazole rings. The major packing difference comes from the mutual orientation/location of the apolar parts, i.e., the ethyl chains leading to complete segregation in α and only isolated apolar channels in β (Figure 1e,f), leading to a ∼2.3% contraction of the unit cell in β.

Forms α and β are not symmetry related; not even the heavy metal atom positions and no direct conversion pathways could be found for the conversion of one form into the other, suggesting that the phase transition is not second order (continuous). While SC–SC transformations are well studied,^12^ SC–SC polymorphic transitions with major packing changes not only are incredibly rare^10,13^ but also suggest a more complex mechanism of transformation involving nucleation and growth,^14^ though avoiding the collapse of the crystal. DFT calculations indicate multiple close local energy minima in the gas phase or solution, suggesting easy transformation between the different conformations and high importance of the intramolecular interactions which certainly are important during crystallization and in the solid state. For the latter a slight (8.8 kJ/mol) energetic preference for the β phase has been calculated (see Figure S5).

**Figure 2:**
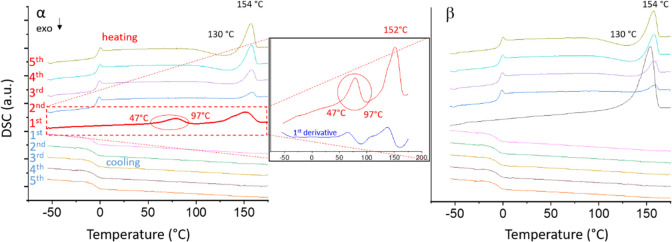
DSC thermograms of α (left, 5 °C/min), β (right, 5 °C/min), and the first heating of α (middle, 1 °C/min) with its first derivative.

To investigate the phase transition further, particularly its order, differential scanning calorimetry (DSC) (Figure 2) was conducted. On heating from −60 to 175 °C, form α displayed a very broad endothermic event taking place from 47 to 97 °C, followed by a second broad endothermic event occurring between 145 and 162 °C. Optical microscopy revealed the latter event to be a melting transition. Upon cooling the melt, only glass formation at −1 °C could be observed. Upon heating devitrification sets in at about the same temperature, followed by cold crystallization right before melting, reminiscent of ionic liquid and glass behavior.^15^ All repeated thermal cycles are identical with the second one and are also identical with the DSC cycles recorded for β. For virgin β samples, a melting point of 154 °C was recorded. The first derivative of the α to β transition (Figure S7) indicates that the phase transition is first order. The endothermic character suggests that both forms are thermodynamically stable under the respective conditions, albeit it appears to be (kinetically) irreversible, since only β crystallizes from the melt.

**Figure 3:**
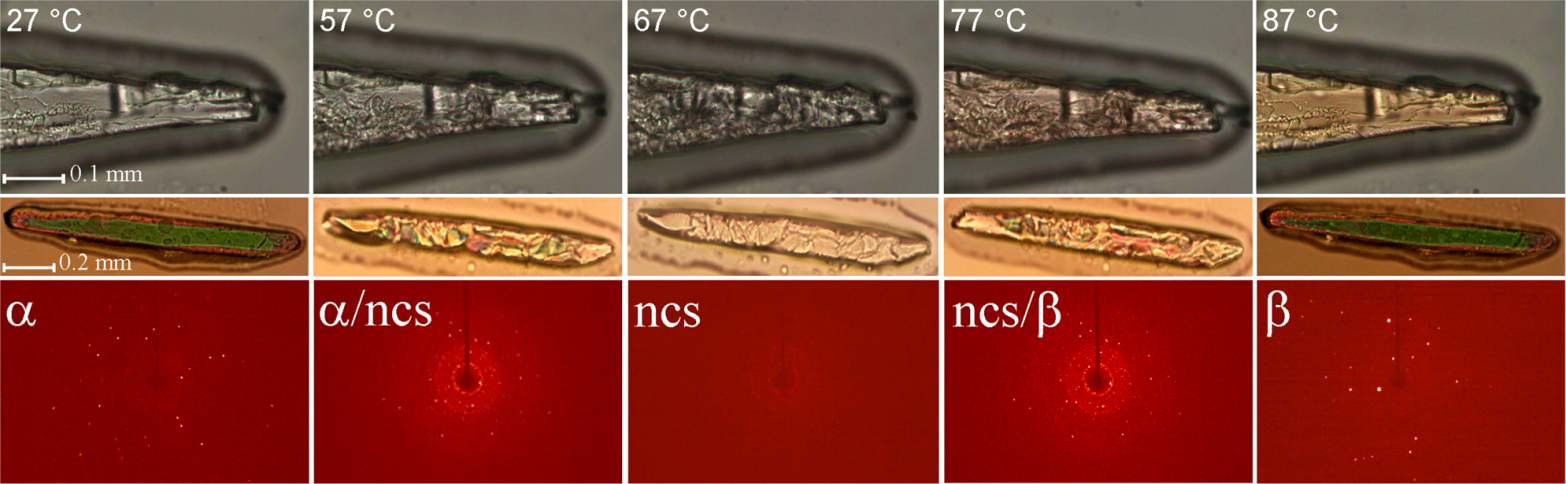
Evolution of the crystal shape, color, and diffraction pattern upon continuous heating starting from α characterized via conventional (top) and polarized (middle) optical microscopy, and SCXRD (bottom) (ncs = noncrystalline solid). The halo around the crystal belongs to the mineral oil used to attach it to the holder.

The rather broad solid–solid phase transition points to a kinetically slow process that is sufficiently slow to be followed by optical microscopy (OM) and laboratory X-ray diffraction (Figure 3). The OM studies were conducted on a heating stage following the same ramps used for the thermal analysis and revealed a gradual disappearance of birefringence for crystals of α (Figure 3 (middle)) starting at the onset of the transition seen in DSC (47 °C). Birefringence is regained starting at ∼77 °C. When observed under unpolarized light, a wave of translucence is observed to propagate through the crystal (Figure 3 (top), Movie S1) that coincides with the loss of birefringence observed under polarization (*T* = 67 °C). Upon continued heating, the wave propagates in reverse and the orange color change is observed at ∼80 °C. The crystal gradually regains transparency and birefringence until it displays a bright orange color at 87 °C. No detectable change in morphology is observable until the melting process sets in at 97 °C (as noted by the beginning of loss of crystallinity detected by SCXRD).

The observation of a SC–amorphous–SC transition is supported by temperature dependent SCXRD (Figure 3 (bottom)). By determining the unit cell at each temperature (every 5 °C and every 1 °C around the transition temperature 60–75 °C), it can be seen that the crystal remains as α up to 57 °C, at which point we observed the first signs of X-ray amorphization. Continued heating caused further loss of crystallinity. At 67 °C the unit cell determination was impossible due to lack of reflections indicating a predominantly X-ray amorphous stage. Crystalline features are then regained at 77 °C where the crystallographic characteristics of β are observed. It is also important to note that although the crystal morphology, i.e., the outer shape, remained visibly unaltered, there is no crystallographic relationship between the expressed crystal facets. Indexing of the exposed crystal facets of a crystalline specimen before and after the phase transformation (see Supporting Information Figure S12).

Slight color changes were observed past the amorphous stage. Once at 87 °C, the crystal exhibited an intense orange color indicative of β. After the transition, the unit cell of β could be easily indexed. At 97 °C, the crystal starts to lose crystallinity as expected for the melting process. It is worth noting that both IR and Raman spectroscopic measurements (Figures S9 and S10) do not show changes in the characteristic vibrations during the transition. Particularly, the Raman spectra show that the complex remains intact as the characteristic Zn–S and Zn–Cl vibrations remain unaltered even in the molten state. The X-ray pair distribution function (PDF) calculated for the intermediate stage presented in Figure S4iv also confirms preservation of the local coordination environment (as only one strong peak can be observed at ∼2.3 Å covering both the Zn–S and Zn–Cl contacts) and the absence of any long-range order (Figure S11).

Phase analysis throughout the experiment using the powder method^16^ indicated a peak value of 92% of the amorphous component in the midst of the observed broad transition, around 67 °C. This observation matches the reversible wave propagating throughout the crystal, slowing down at the inversion point (Figure 3 (top), Movie S1) and the significant decrease of the diffraction spots (Figure 3 (bottom)). Astonishingly, the X-ray amorphous stage can be stabilized for numerous hours if the applied heating is halted (or the crystal cooled to room temperature). The transition will proceed further only when heating is resumed and the temperature reaches where it was prior to cooling. This confirms the previous observation that the transition is kinetically slow.

Despite the fact that *the crystals in each study retained the same shape and physical appearance, down to the same cracks and surface defects*, a conventional SC–SC transition^17^ can be ruled out. Instead, α converts to β via an amorphous state (γ) which can be maintained for an extended time unless heated further. This raises the question of how the crystals can retain their shape and surface detail in a SC–amorphous–SC transformation.

As noted earlier, the conversion of α to β requires the thione ethyl groups and the thiones themselves to reorient, which would require sufficient energy to give rotational freedom and allow translational freedom for the Zn complexes (see Figure S6). We hypothesize that freely rotating ethyl groups and thione rings could act as a “glue” providing a rigid glassy state, holding the tetrahedra formed by S–Zn–Cl, and allowing slow recrystallization into β while maintaining the morphology of the crystal. This would be supported by the wave-like propagation of crystalline-to-amorphous and amorphous-to-crystalline observed by microscopy.

## Conclusion

In summary, what appeared to be just another example of a traditional SC-to-SC shape preserving polymorphic transition of a simple coordination compound was revealed to be anything but traditional and quite intricate. The unique nature of the ZnCl_2_(C_2_C_2_ImT)_2_ complex provided kinetically slow transformations between the polymorphs which could be followed, providing clear evidence of a first order SC–amorphous–SC transformation with shape preservation. We believe this observation is more common than current literature would suggest, and our finding may have more relevance to the broader field and will stimulate deeper investigations on the solid–solid phase transformations. Since this transition requires essentially just a flexible ligand being able to adopt its shape in order to support the backbone of the structure, we suspect that many other purported SC–SC and, in a wider sense, solid–solid phase transformations may also proceed through a solid amorphous phase.

## Supporting Information Available

Additional experimental details, materials and methods, UV–vis absorption and photoemisson spectra, ^1^H and ^13^C NMR spectra, PXRD patterns, PDF analysis, results of DFT calculations, addional crystallographic details, thermogravimetric analyses, additional SCXRD analyses, FT-IR and Raman spectra, Figures S1–S12, and Table S1 (PDF)
Movie S1 of α to β transition (MP4)

## Accession Codes

CCDC 1965743–1965744 contain the supplementary crystallographic data for this paper. These data can be obtained free of charge via www.ccdc.cam.ac.uk/data_request/cif, or by emailing data_request@ccdc.cam.ac.uk, or by contacting The Cambridge Crystallographic Data Centre, 12 Union Road, Cambridge CB2 1EZ, UK; fax: +44 1223 336033.
