# Supplementary material for: Shape Preserving Single Crystal to Amorphous to Single Crystal Polymorphic Transformation Is Possible
Source: J Am Chem Soc. 2021 Nov 23;143(48):20202–6. doi: 10.1021/jacs.1c08590 (PMC8662720; doi:10.1021/jacs.1c08590)
Supplement: Supplementary file 1 — ja1c08590_si_001.pdf [file ja1c08590_si_001.pdf]

Shape Preserving Single Crystal-to-Amorphous-to-Single Crystal Polymorphic Transformation Is Possible

Olivier Renier[#], Guillaume Bousrez, Glib V. Baryshnikov, Veronica Paterlini, Volodymyr Smetana[#], Hans Ågren, Robin D. Rogers[*], and Anja-Verena Mudring[*]

Correspondence to: anja-verena.mudring@mmk.su.se, rdrogers@ua.edu

Supporting Information

This file includes:

Materials and Methods
Supplementary Text
Figures S1 to S12
Table S1
Caption for Movie S1

Other Supplementary Materials for this manuscript include the following:

Movie S1

Materials and Methods

Reagents were used as obtained from commercial sources (Merck KGaA, Darmstadt, Germany). All solvents were 'solvent grade' and used as received without additional purification. Basic synthetic routes are provided in Figure S1.

Synthesis of 1-ethylimidazole

1-H-imidazole (50 mmol, 1.0 eq.) and potassium carbonate (101 mmol, 2.02 eq.) were dissolved in tetrahydrofuran (50 mL). Ethyl iodide (55 mmol, 1.1 eq.) was added and the mixture was stirred during 3 h at room temperature. The solution was filtered and the filtrate was concentrated and the residue dried under vacuum.

Synthesis of 1,3-diethylimidazolium bromide [C_2C_2Im][Br]

1-Ethylimidazole (50 mmol, 1.0 eq.), ethyl bromide (55 mmol, 1.1 eq.), and acetonitrile (10 mL) were heated under reflux for three days. After cooling to room temperature the solution was washed with ethyl acetate and put in a cold bath (-78 °C) to allow the crystallization of the product. The white precipitate was washed multiple times with ethyl acetate and then dried under vacuum at room temperature.

Synthesis of 1,3-diethyl-imidazole-2-thione (C_2C_2ImT)

The imidazole-2-thione compound was synthesized based on the procedure found in the literature.[1] 1,3-Diethylimidazolium bromide [C_2C_2Im][Br] (10.2 g, 50.0 mmol, 1.0 eq.), potassium carbonate (8.28 g, 60.0 mmol, 1.2 eq.), elemental sulfur (1.66 g, 6.5 mmol, equivalent to 52.0 mmol of sulfur atoms, 1.04 eq.) and methanol (50 mL) were introduced in a round-bottomed flask. The mixture was heated at 70 °C for 48 h. The solvent was then removed under reduced pressure. The crude was extracted with dichloromethane and water. The organic phase was dried with magnesium sulphate and concentrated using a rotary evaporator. A dark brown solid was obtained with a yield of 54 % (4.22 g). ^{1}H-NMR (400 MHz, $CDCl_3$): 1.11 (t, $J_{H\text{-}H}$ = 7.2 Hz, 6H), 3.84 (q, $J_{H\text{-}H}$ = 14.4 Hz, $J_{H\text{-}H}$ = 7.2 Hz, 4H), 6.53 (s, 2H). ^{13}C-NMR (100 MHz, $CDCl_3$): 14.0, 42.4, 115.9, 160.5. ν_{max} (cm^{-1}): 3155, 3116, 3081, 2976, 2962, 2934, 2874, 2675, 1667, 1568, 1517, 1462, 1449, 1412, 1382, 1365, 1349, 1321, 1273, 1256, 1212, 1158, 1105, 1088, 1049, 1031, 947, 913, 835, 790, 722, 713, 680, 631, 617, 514.

Bis(1,3-diethylimidazole-2-thione)zinc(II) chloride ($ZnCl_2(C_2C_2imT)_2$).

Form *α*: A mixture of 1,3-diethylimidazole-2-thione (156 mg, 1.0 mmol, 2 eq.) and $ZnCl_2$ (68 mg, 0.5 mmol, 1 eq.) were mixed in 5 mL water. The solution was stirred for 4 h at room temperature. We obtained a clear, colorless solution. It was then allowed to stand at room temperature. Slow evaporation gave colorless mm-sized crystals (Figure S2) within a few days with a quantitative yield as confirmed by SCXRD and NMR (Figure S3).

Form *β*: The same procedure as for form *α* was followed but the obtained solution was evaporated by means of a rotary evaporator at 40 °C. The obtained solid was bright orange crystalline powder. An additional method of obtaining this form is by mechanically grinding the colorless crystals obtained from the synthesis of the form *α*.

^{1}H-NMR (400 MHz, $CDCl_3$): 1.41 (t, $J_{H\text{-}H}$ = 7.2 Hz, 12H), 4.23 (q, $J_{H\text{-}H}$ = 14.4 Hz, $J_{H\text{-}H}$ = 7.2 Hz, 12H), 6.96 (s, 4H). ^{13}C-NMR (100 MHz, $CDCl_3$): 14.6, 43.9, 118.7, 152.4. ν_{max} (cm^{-1}): 3152, 3120, 3100, 2979, 2934, 2874, 1614, 1564, 1525, 1476, 1454, 1419, 1384, 1351, 1266, 1207, 1161, 1116, 1089, 1052, 1034, 951, 797, 754, 739, 711, 685, 663, 624, 607, 504, 412

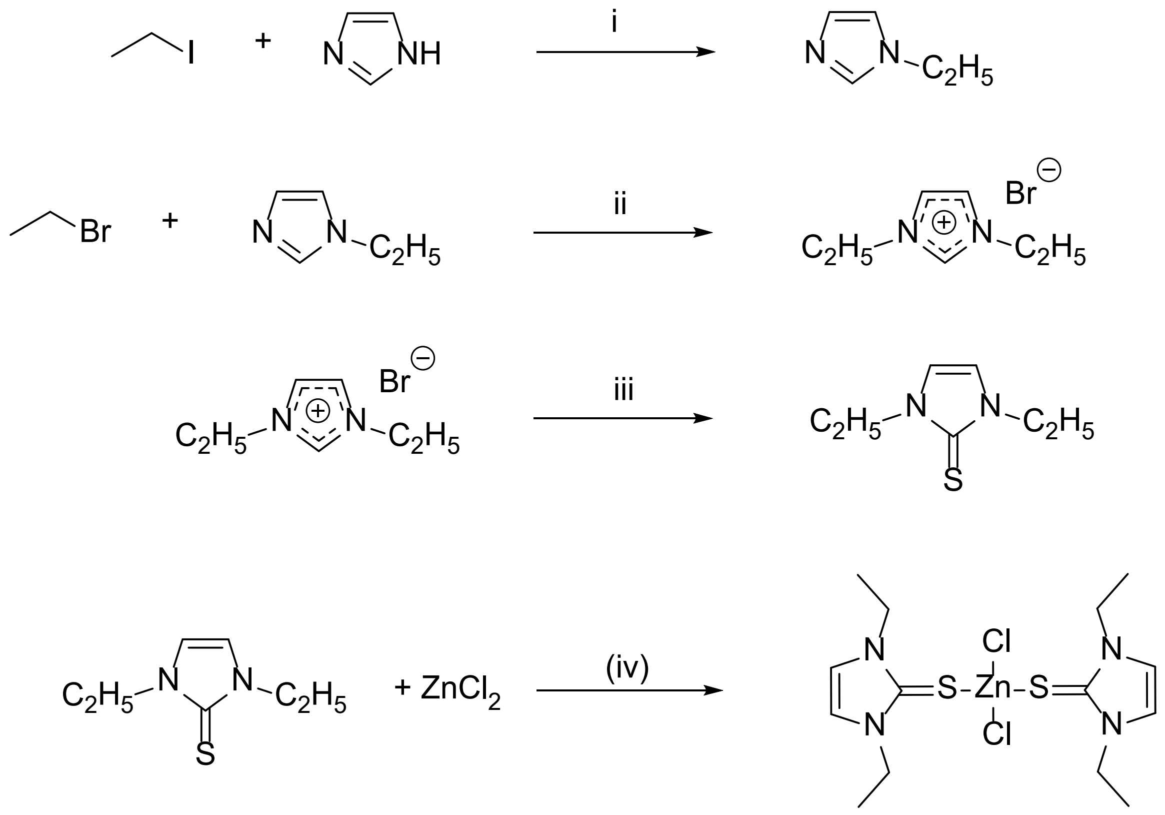

Figure S1. Syntheses of imidazole, imidazolium, imidazole-2-thione, and complexes: i) THF,

K_2CO_3, room temperature, 3 h; ii) acetonitrile, reflux, 72 h; iii) methanol, K_2CO_3, S_8, reflux; (iv) H_2O, room temperature, 4 h.

Instrumentation

Thermogravimetric Analysis (TGA) was performed with a TG 449 F3 Jupiter (Netzsch, Selb, Germany) analyzer. Measurements were carried out in aluminum oxide crucibles with a heating rate of 10 °C/min and nitrogen as purge gas.

Differential scanning calorimetry (DSC) was performed with a computer-controlled PhoenixDSC 204 F1 thermal analyzer (Netzsch, Selb, Germany). Measurements were carried out at a heating rate of 5 °C/min in sealed aluminum crucible with an argon flow rate of 40 mL/min. The samples were placed in aluminum pans which were cold-sealed. The samples were first cooled to -60 °C without recording any data and then heated up from this point. The α-β transition was also investigated at 1 °C/min.

Infrared spectroscopy (IR) was conducted with a Bruker Alpha-P ATR-spectrometer (Karlsruhe, Germany) in attenuated total reflection configuration. The data evaluation was carried out with the program OPUS (Bruker, Ettlingen, Germany).

^1H and ^{13}C NMR spectra were recorded at room temperature in $CDCl_3$ on a Bruker 400 MHz spectrometer equipped with a BBO probe (Karlsruhe, Germany). Chemical shifts are reported in delta (δ) units, expressed in parts per million (ppm). The following abbreviations used for the observed multiplicities: s (singlet), d (doublet), t (triplet), q (quartet), m (multiplet for unresolved lines). ^1H NMR chemical shifts were referenced to the residual solvent signal for $CDCl_3$ (7.26 ppm) and ^{13}C NMR chemical shifts were referenced to the solvent signal of $CDCl_3$ (77.16 ppm).

The ESI mass spectrometry was conducted with A SYNAPT G2-S HDMS Q-ToF Mass Spectrometer (Waters, Manchester, UK) in positive ion mode.

Single crystal X-Ray diffraction data were recorded using a Bruker D8 Venture (Mo Kα, at 293 K) (Karlsruhe, Germany). The crystals were mounted on a glass fiber or cryogenic loop with the help of a minor amount of mineral oil or Apiezon® grease. Crystal structure solution by direct methods using SHELXT[2] yielded the heavy atom positions. Refinement with SHELXL[3] allowed for the location of the remaining atom positions. Hydrogen atoms were added and treated with the riding atom mode. Data reduction was performed with the program package X-Red or SAINT and

absorption corrections were carried out with the programs X-Shape or SADABS.[4-5] To illustrate the crystal structures, the programs Diamond and Mercury were used.[6-7]

Temperature dependent Single crystal X-Ray diffraction studies were conducted on the same instrument using an Oxford instrument liquid nitrogen cooling system. The heating rate was 5 °C/min. Frames were taken every 2 °C with a stabilization time of 5 min.

The amorphous stage stability was tested by heating of a α crystal until the diffraction spots had disappeared. From this point on, the crystal was back down to room temperature while still taking diffractogram at 5 °C intervals. The crystal was then left on the mounting loop overnight and the heating was resumed the next day.

Optical analyses were made by heated-stage polarized optical microscopy (POM) with an Axio Imager A1 microscope (Carl Zeiss MicroImagingGmbH, Göttingen, Germany) equipped with a hot stage, THMS600 (Linkam Scientific Instruments Ltd, Surrey, UK), and Linkam TMS 94 temperature controller. Images were recorded at a magnification of 50× as a video with a digital camera. Heating and cooling rates were 5 °C/min.

Powder X-Ray diffraction data were recorded at ambient temperature on a PANalytical X'pert PRO diffractometer (Malvern Panalytical, Malvern, UK), operating at 45 kV and 40 mA and using $CuK_{\alpha 1}$ radiation. The data were recorded in reflection mode from 5° to 70° with a step size of 0.01° for 60 min.

Steady-state fluorescence excitation and emission spectra were recorded on a HORIBA Jobin Yvon FluoroLog-3 modular spectrofluorometer, equipped with a 450 W xenon arc lamp and an R928P PMT detector (Horiba France, Longjumeau, France).

The UV-Vis diffuse reflectance spectrum was recorded on a powder sample made from the obtained crystals using an Agilent Technologies Cary 5000 UV-Vis-NIR spectrophotometer equipped with an Agilent Praying Mantis diffuse reflectance accessory. A Spectralon disk was used as the reference material for the measurements on powder and crystals (Agilent Technologies, Kista, Sweden).

Computational work

Gas phase

After geometry optimization, the electronic structure of the complex was calculated using the Gaussian 16 package[8] using the B3LYP/6-31G(d,p) method with accounting of Grimme's D3

empirical correction dispersion in the gas phase approximation. The initial models were taken from the SCXRD data. Based on the S_0 state optimized geometries the vertical absorption spectra were calculated within time-dependent (TD) DFT method using the same GD3-B3LYP/6-31G(d,p) approximation with and without accounting for solvent effects. The polarizable continuum model (PCM) was used for the simulation of a solvent environment, acetonitrile was chosen to be able to compare to experimental absorption and emission spectra. In order to explain the effect of crystal packing on the absorption spectra of the studied ***α*** and ***β*** forms cut-outs containing 6 and 12 molecules from the extended structure obtained from X-ray diffraction analysis were used. For the clusters containing 6 molecular units the topological analysis of electron density distribution function was additionally performed by Bader's "Atoms in Molecules" (AIM) method.[9] These calculations allow to predict the presence and strength of all intermolecular interactions (H-bonds, π-π staking, etc.) within selected crystal fragments and thus one can conclude about the effect of intermolecular interactions on the quite different optical properties of the ***α*** and the ***β*** forms. Finally, the emission (fluorescence) spectra of the studied ***α*** and ***β*** forms of the complex and free ligand molecule were calculated based on optimization of first excited singlet (S_1) state at the TD-DFT/GD3-B3LYP/6-31G(d,p) level of theory with and without accounting of solvent effect. Geometry optimization and spectra calculations were performed within Gaussian 16 software, while AIM analysis was carried out by using AIMAll program package.[10]

Solid state

To understand the phase relationship between the ***α*** and the ***β*** forms calculations for the extended solids have been performed. Full structural optimizations and band structure calculations were carried out with the projector-augmented wave (PAW) method of Blöchl[11] as implemented in the *Vienna ab initio Simulation Package* (VASP) by Kresse and Joubert.[12-16] Correlation and exchange were described by the Perdew-Burke-Enzerhof general gradient approximation (GGA−PBE).[17] Starting meshes of $4 \times 4 \times 4$ *k*-points for both compounds were applied to sample the first Brillouin zones for reciprocal space integrations, while the energy cut-offs of the plane-wave basis sets were set to 500 eV. With these settings, the calculations converged until the energy difference between two iterative steps fell below 10^{-7} eV/cell.

The phase transition can also be visibly followed through its color change because the two polymorphs show different absorption (**Figure S2**), originating from the different crystal packing as confirmed by time-dependent DFT analysis. Absorption bands in the UV region (200-300 nm) can be assigned to the $S_0 \rightarrow S_1$ and $S_0 \rightarrow S_2$ transitions corresponding to π–π* and n–π* electronic states of the imidazolium ring in $ZnCl_2(C_2C_2ImT)_2$, respectively (**Figure S5**) The broad band at lower energies (~400–600 nm) originates from intermolecular interactions, as it corresponds to the electronic charge-transfer transitions between occupied molecular orbitals of one unit and unoccupied molecular orbitals of the neighboring complexes. For the β form, this transition is significantly more intense and red-shifted with respect to the α form, giving rise to its orange color (**Figure S2**).

In solution, all possible conformations including intermediate more stable forms can exists, and indeed, there is no absorption in the visible region. The main absorption band is observed around 270 nm for the complex, as well as, for the ligand (**Figure S2**), which mostly overlaps with the absorption of the CH_3CN species. As expected, the optical properties in CH_3CN solution are quite similar whether prepared from α or β crystalline forms since the crystalline packing is lost during dissolution. The lack of absorption in the visible region confirms that the orange color of β observed in the solid state derives from its crystal packing.

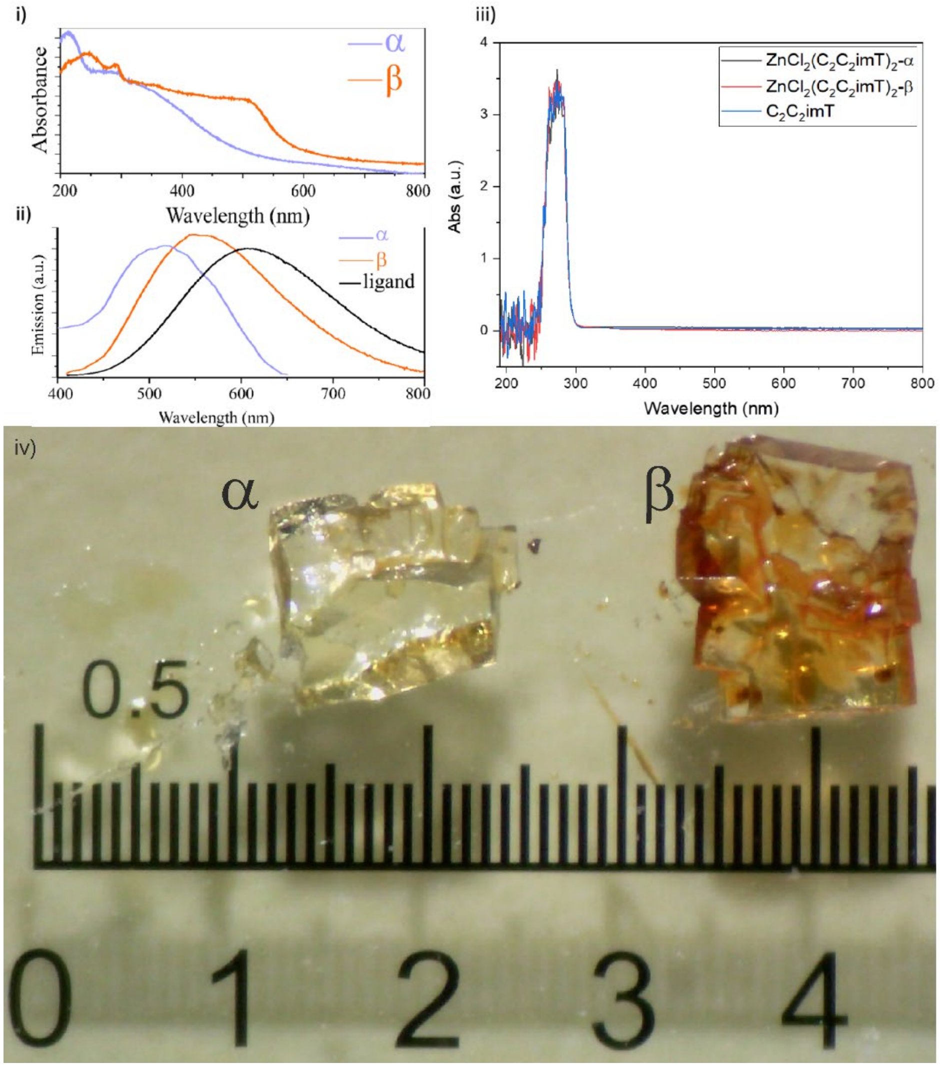

Figure S2. i) UV-vis absorption spectra collected from solid samples ***α*** and ***β*** showing the difference in color between the two compounds; **ii)** Photoemission spectra (a.u.) of C_2C_2ImT, ***α*** and ***β*** in the solid state (powders for ***β*** and the ligand and crystals for ***α***). Excitation wavelength for all samples = 288 nm; **iii)** Absorbance spectra of ***α***, ***β***, and C_2C_2imT in acetonitrile solutions; **iv)** crystals of ***α*** and ***β***.

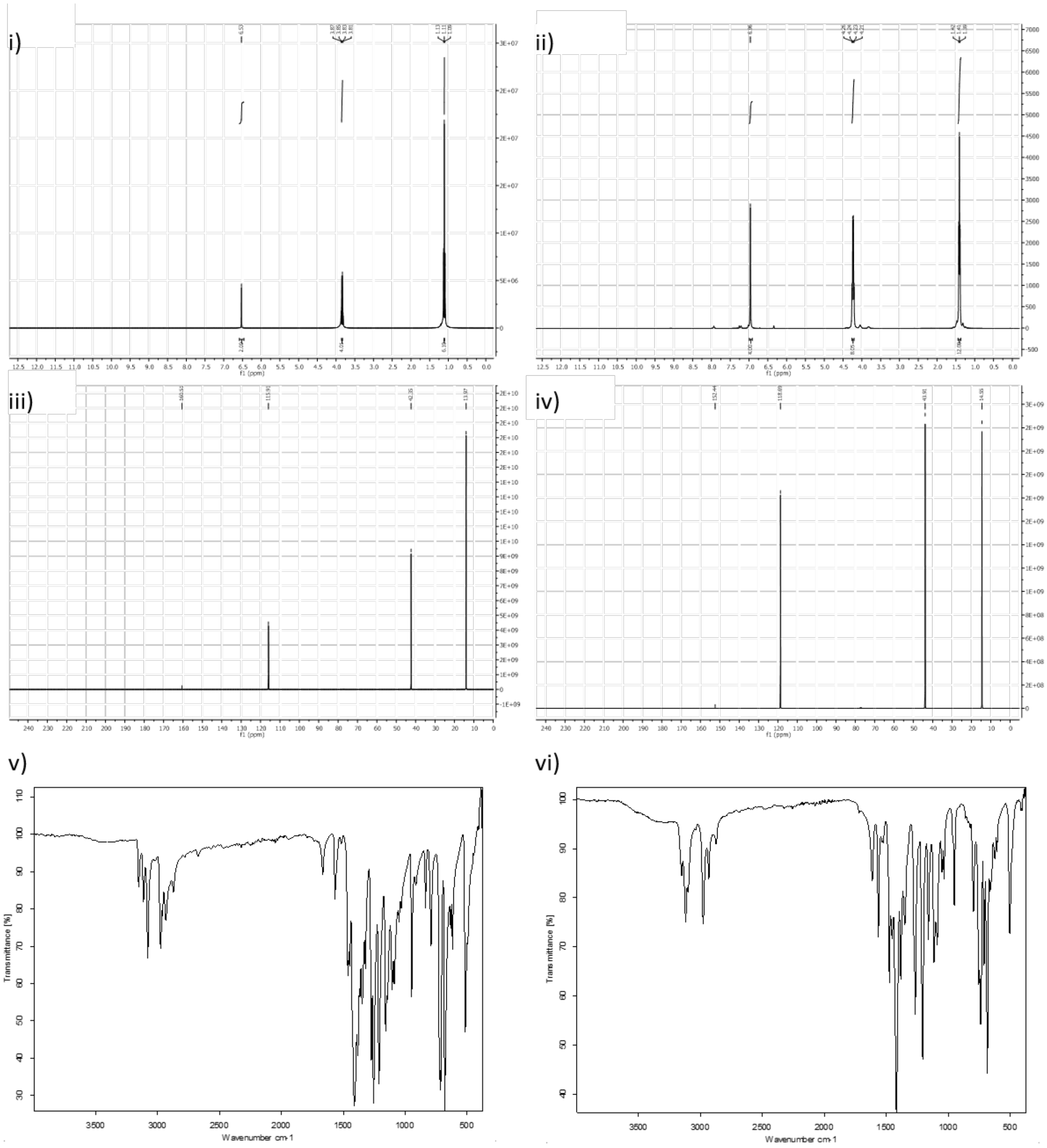

Figure S3. i) ^{1}H NMR spectrum of C_2C_2ImT; **ii)** ^{1}H NMR spectrum of $ZnCl_2(C_2C_2ImT)_2$; **iii)** ^{13}C NMR spectrum of C_2C_2ImT; **iv)** ^{13}C NMR spectrum of $ZnCl_2(C_2C_2ImT)_2$ in $CDCl_3$; **v)** ATR FT-IR spectrum of C_2C_2ImT; **vi)** ATR FT-IR spectrum of ***β***.

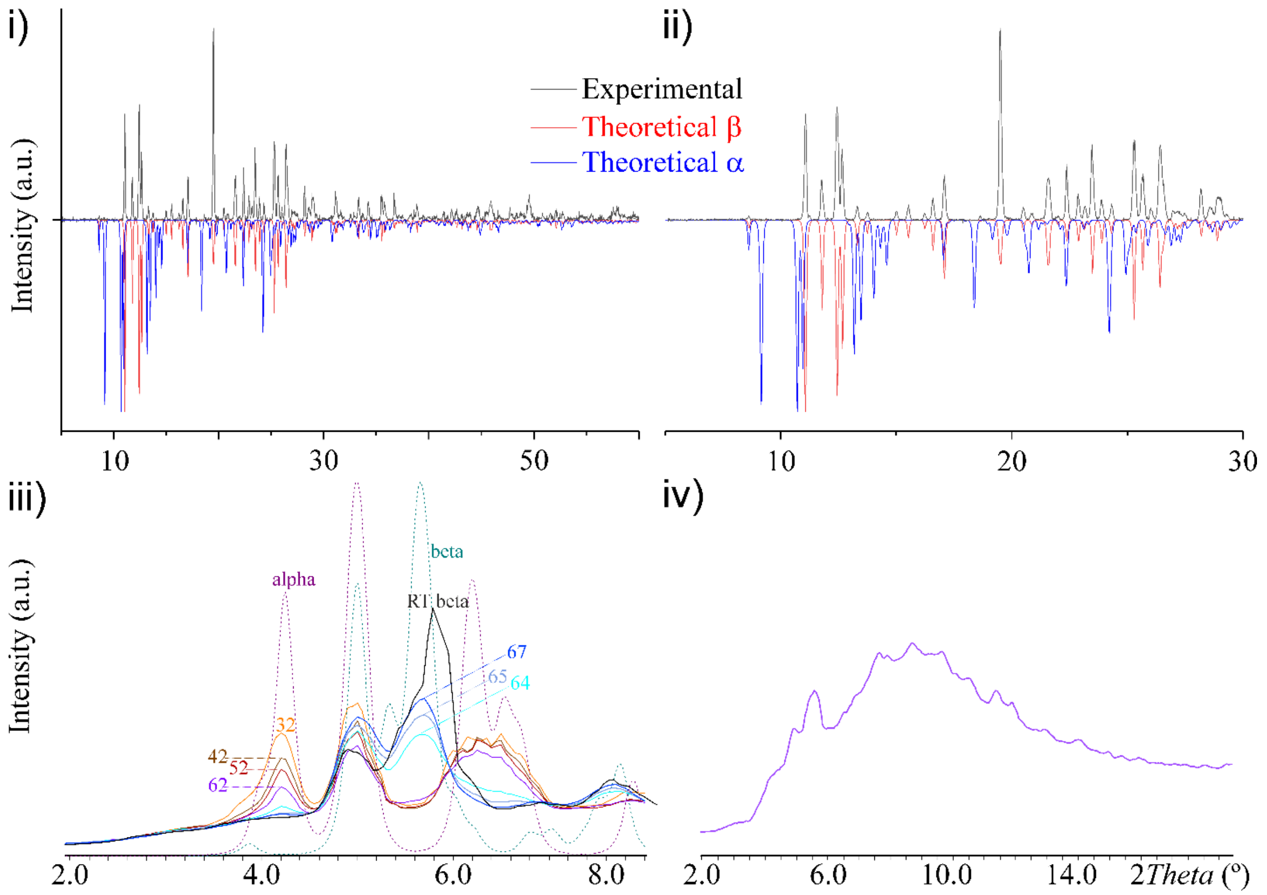

Figure S4. i) PXRD pattern (Cu $K_{\alpha 1}$) of crushed Form $\boldsymbol{\alpha}$ (*black line*) compared to the calculated PXRD pattern from the Form $\boldsymbol{\beta}$ (*red line*) and Form $\boldsymbol{\alpha}$ (*blue line*) crystal structures; **ii)** Magnified version of the PXRD pattern between 5 and 30°; **iii)** Evolution of the PXRD pattern of the crystal of **α** upon heating with extended (5–15 minutes) annealing at each point (dashed purple = theoretical $\boldsymbol{\alpha}$; dashed green = theoretical $\boldsymbol{\beta}$; solid black line = $\boldsymbol{\beta}$ at room temperature; solid orange = 32 °C; solid brown = 42 °C ; solid red = 52 °C; solid purple= 62 °C; solid cyan = 64 °C solid light blue = 65 °C and solid dark blue = 67 °C); **iv)** PXRD pattern of the crystal of **α** upon continuous heating during the transition stage at ~63 °C.

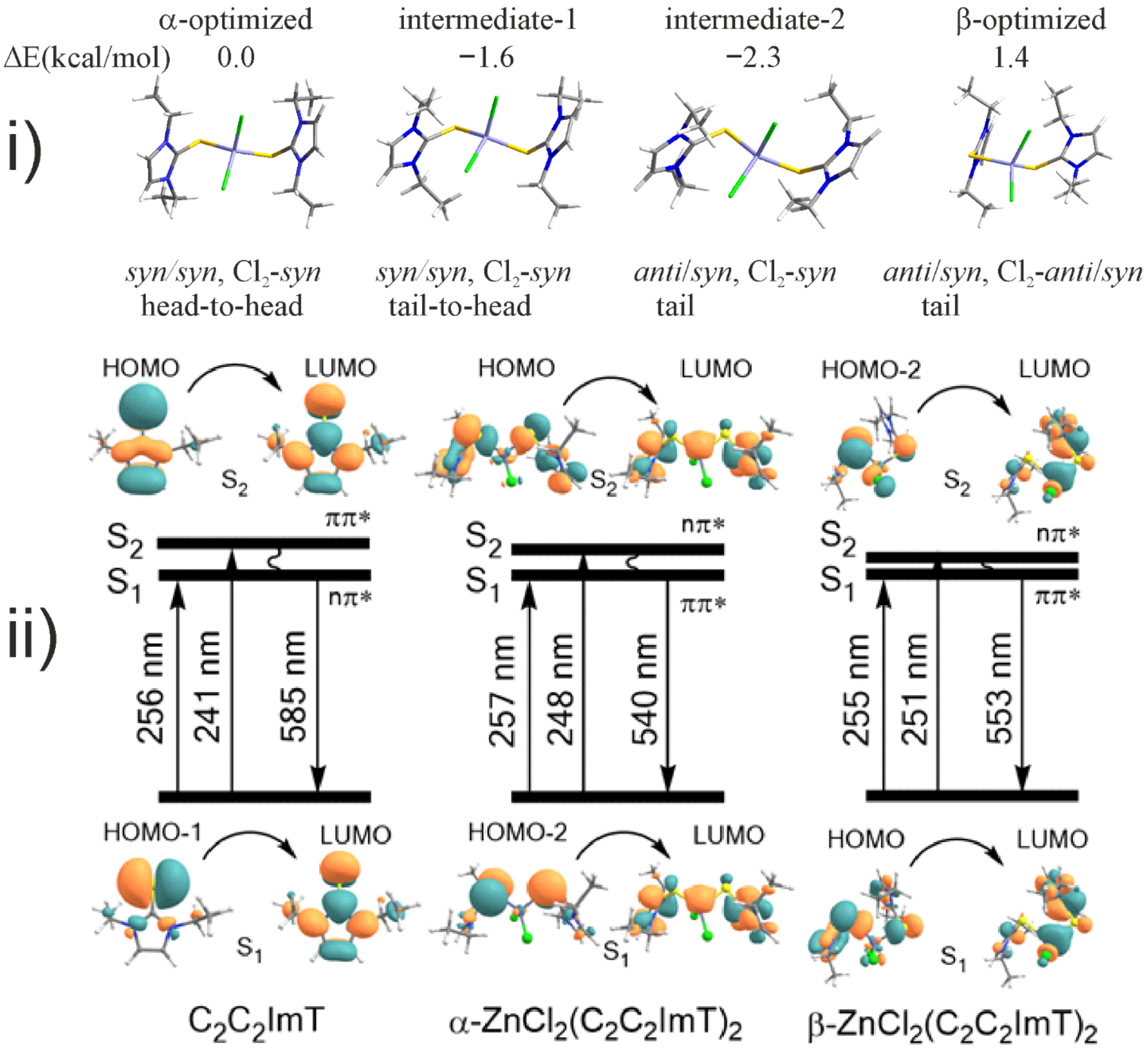

Figure S5. i) The gas-phase DFT-optimized configurations of -$ZnCl_2(C_2C_2ImT)_2$; **ii)** Simplified Jablonski diagram for singlet-singlet absorption and emission processes in C_2C_2ImT, ***α***-$ZnCl_2(C_2C_2ImT)_2$ and ***β***-$ZnCl_2(C_2C_2ImT)_2$ molecules calculated by TD-DFT/GD3-B3LYP/6-31G(d,p) method with accounting of acetonitrile solvent within PCM model.

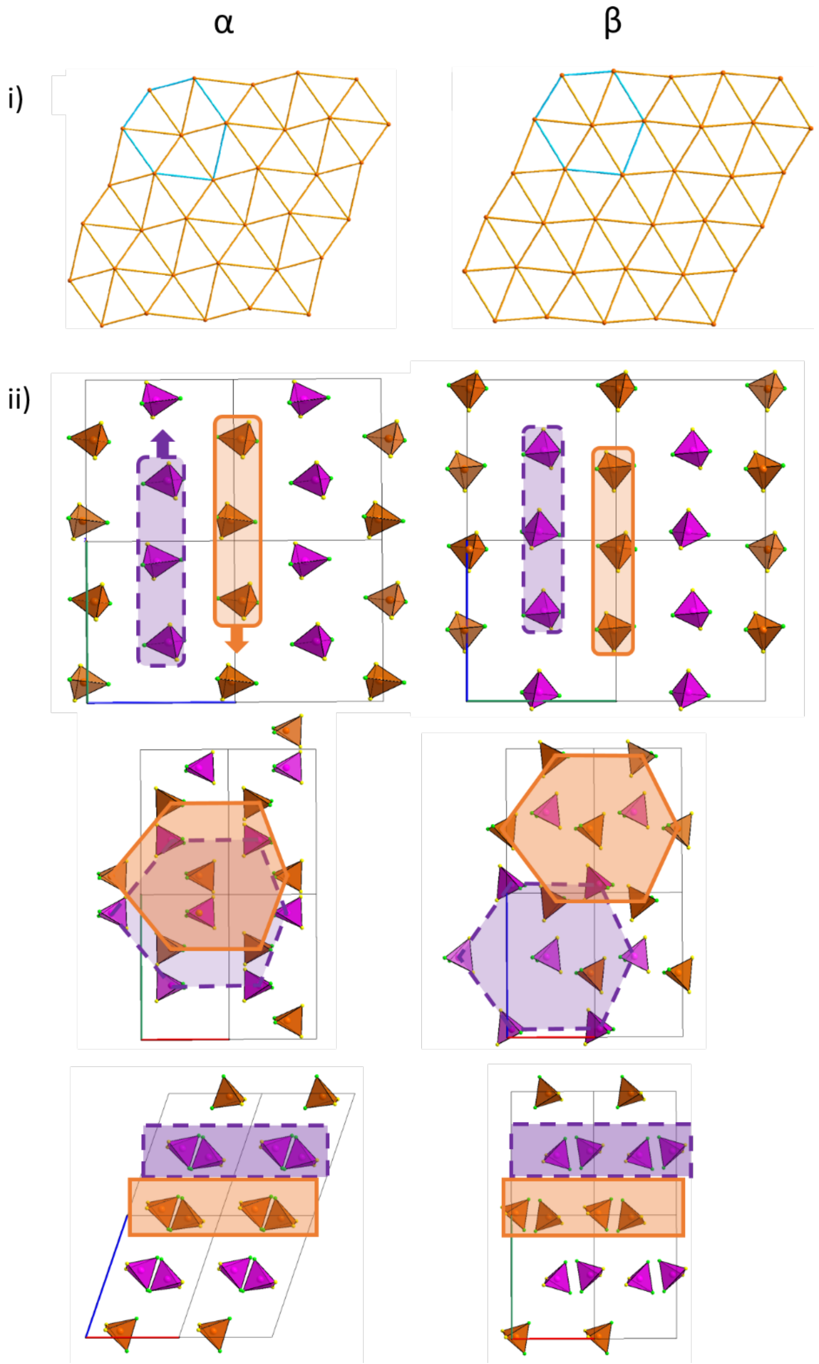

Figure S6. i) Transformation of the Zn lattices upon transition from α to β; **ii)** visual representation of the changes between α and β; same colored polyhedral lie on the same plane; the orange polyhedral are located in front of the purple ones; Element color codes: orange = Zinc; yellow = Sulfur; green = Chlorine; Crystallographic axis are color coded: *a* = red, *b* = green, *c* = blue.

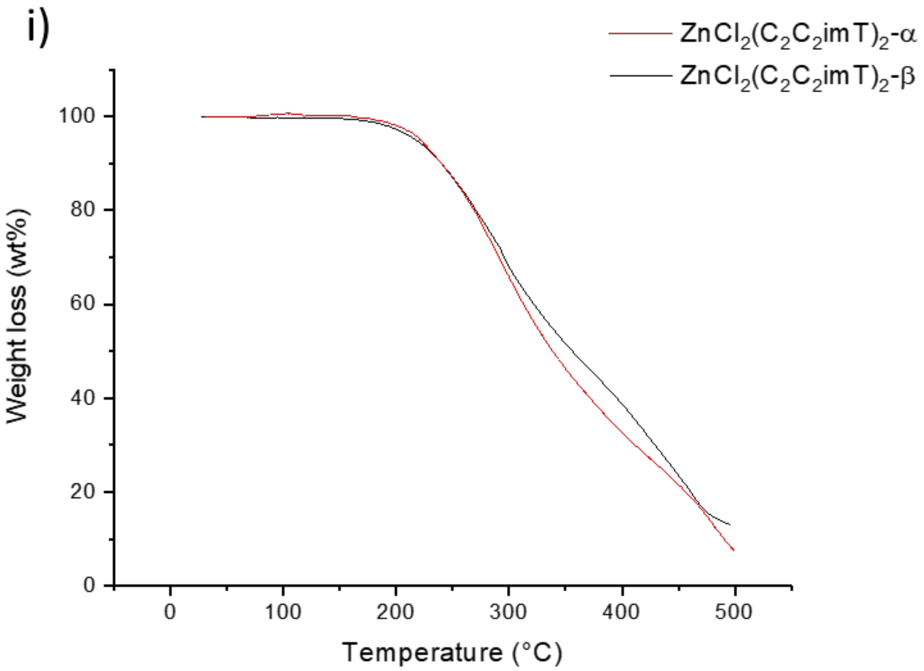

Figure S7. i) Thermogravimetric analysis of α and β (10 ºC/min); T_{onset} = 218.15 °C.

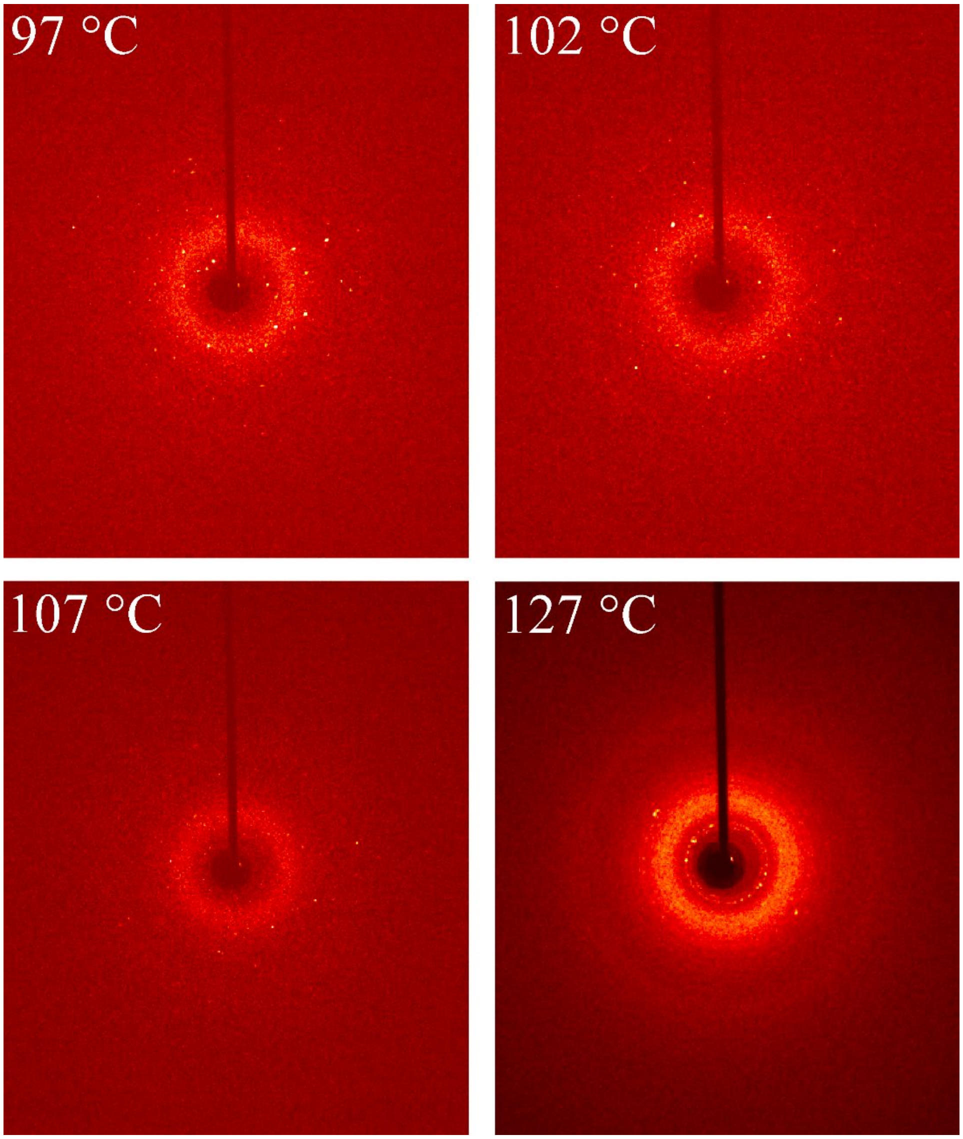

Figure S8. Evolution of the diffraction pattern upon continuous heating across the α to β transition.

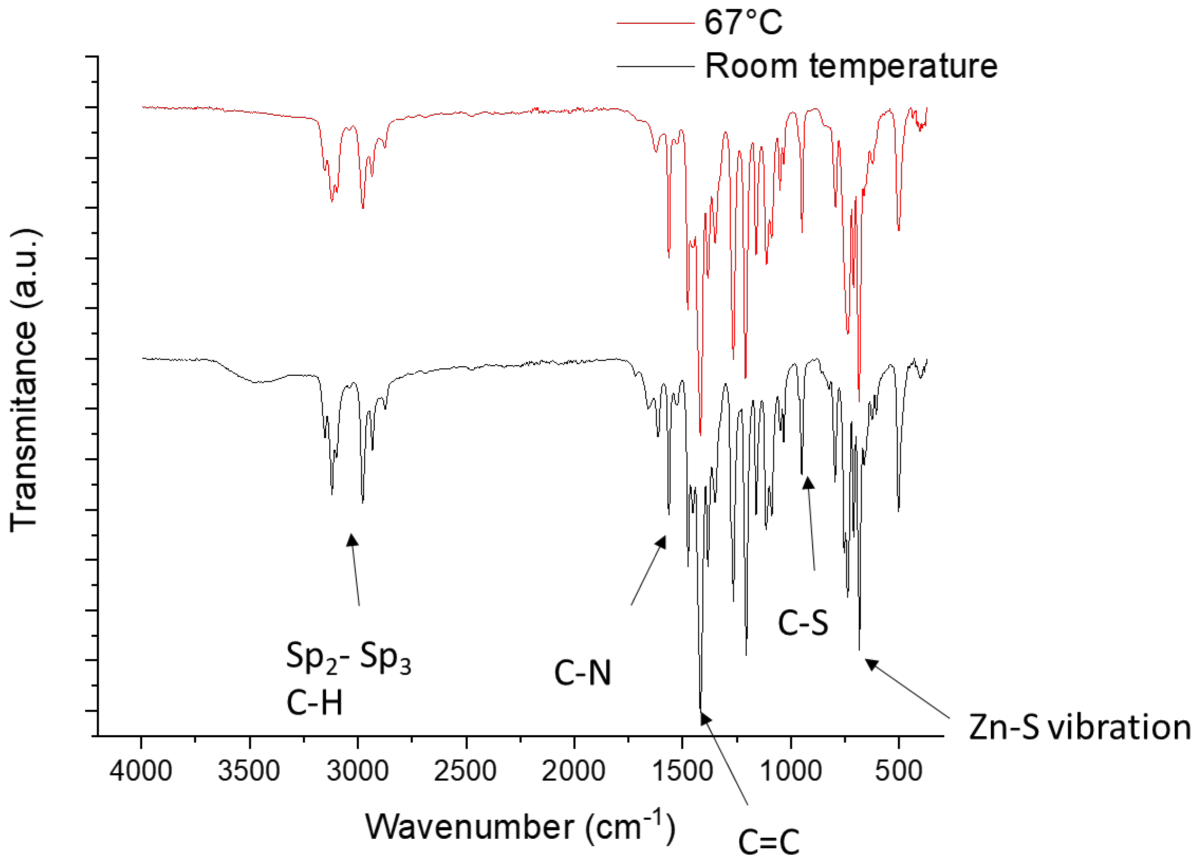

Figure S9. ATR FT-IR spectra of $ZnCl_2(C_2C_2ImT)_2$ collected at a sample of the α-polymorph at RT and during the transition.

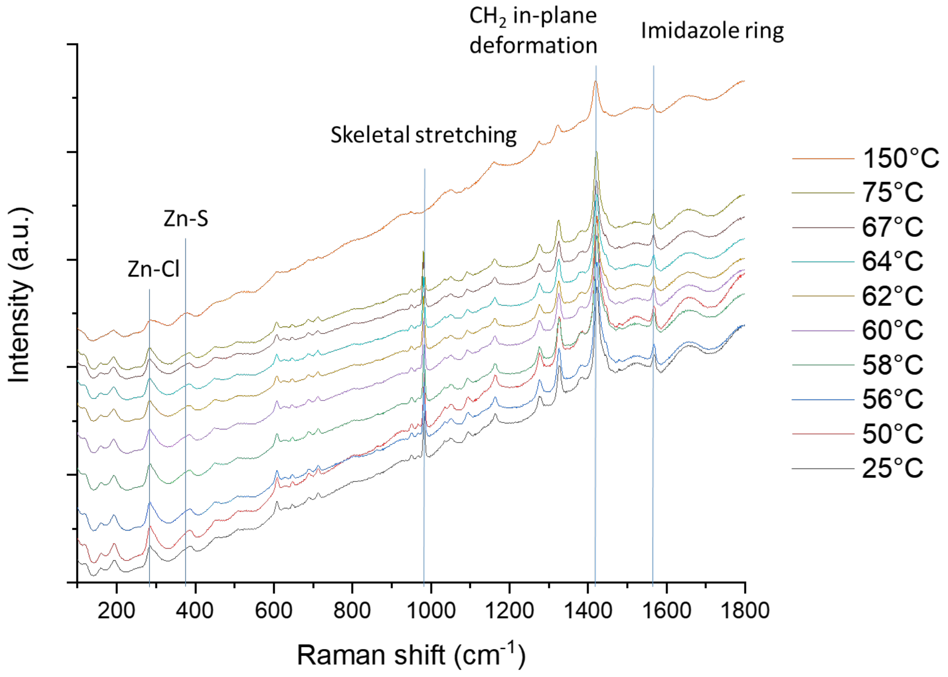

Figure S10. Raman spectra collected on a sample of the α-modification of $ZnCl_2(C_2C_2ImT)_2$ going through the α to β phase transition and melting upon heating.

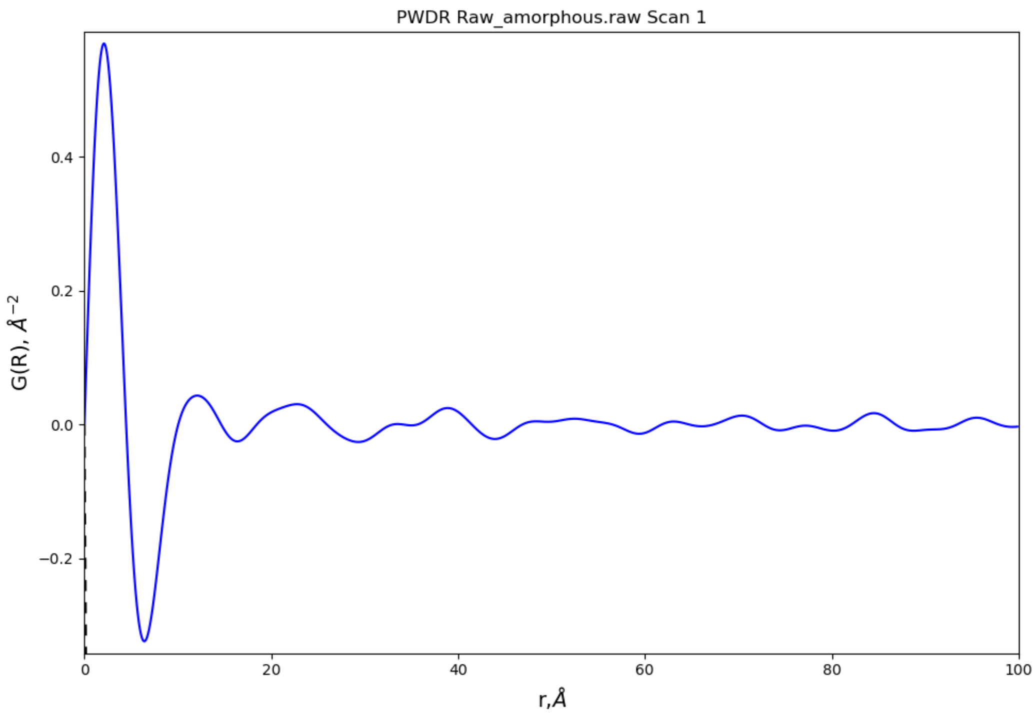

Figure S11. X-ray pair distribution function (PDF) of the intermediate stage presented in Figure S4-iv.

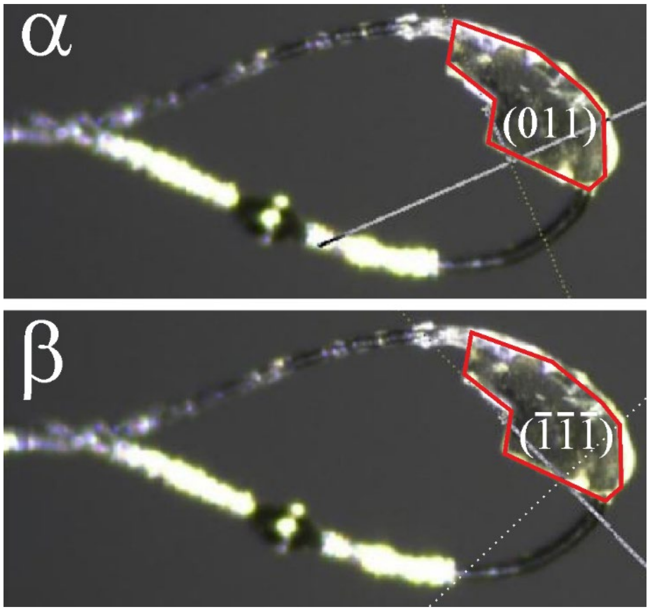

Figure S12. A single crystalline specimen of $ZnCl_2(C_2C_2ImT)_2$ before (α) and after (β) the transformation. The the main crystal facet (parallel to the presentation plane) remains visibly the same, but can be indexed as (011) for the α polymorph and as (-1-1-1) for the β polymorph.

Table S1. Comparison of the characteristic structural details of the different polymorphs.

$ZnCl_2(C_2C_2imT)_2$	α	β
**CCDC**	1965743	1965744
SG	*Pc*	$P2_12_12_1$
***a*, Å**	10.174(1)	9.623(1)
***b*, Å**	15.900(3)	14.463(2)
***c*, Å**	14.160(2)	15.266(2)
**β, °**	108.320(5)	90
***V*, Å³**	2174.5(6)	2124.7(4)
Z	4	4
***T* (°C)**	20	20
**Density (g/cm³)**	1.370	1.403
$d_{Zn–Cl}$, **Å**	2.244–2.264(1)	2.235–2.241(2)
$d_{Zn–S}$, **Å**	2.345–2.366(3)	2.351–2.352(2)
**∠(Im–Im)*, °**	140.6(1)<br>141.3(1)	57.4(4)
$d_{Cg–Cg(intra)}$, **Å**	8.4135(5)<br>8.4251(5)	6.1365(5)
$d_{Cg–Cg(inter)}$, **Å**	4.2653(3)	3.8823–3.8902(2)

Movie S1. Optical microscopy monitoring of α to β transition recorded in a temperature range from 45 °C to 155 °C.

References:

1. Benac, B.; Burgess, E.; Arduengo, A.; Brittelli, D.; Buriak Jr, J.; Smart, B., 1, 3-Dimethylimidazole-2-thione. *Organic Syntheses* **1990,** *7*, 195.
2. Sheldrick, G. M., SHELXT – Integrated space-group and crystal-structure determination. *Acta Crystallographica Section A: Foundations and Advances* **2015,** *71* (1), 3-8, 10.1107/S2053273314026370.
3. Sheldrick, G. M., Crystal structure refinement with SHELXL. *Acta Crystallogr. Sect. C: Struct. Chem.* **2015,** *71* (1), 3-8, 10.1107/S2053229614024218.
4. SAINT, B. A. I., SAINT, Bruker AXS Inc., Madison, Wisconsin, USA, 2015. **2015**.
5. Bruker, M., APEX3 and SADABS. *Bruker AXS Inc., Wisconsin, USA* **2016**.
6. Brandenburg, K.; Putz, H., DIAMOND: Program for Crystal and Molecular Structure Visualization. *Crystal Impact GbR, Bonn, Germany* **2011**.
7. Macrae, C. F.; Edgington, P. R.; McCabe, P.; Pidcock, E.; Shields, G. P.; Taylor, R.; Towler, M.; Streek, J., Mercury: visualization and analysis of crystal structures. *J. Appl. Crystallogr.* **2006,** *39* (3), 453-457.
8. Frisch, M. J.; Trucks, G. W.; Schlegel, H. B.; Scuseria, G. E.; Robb, M. A.; Cheeseman, J. R.; Scalmani, G.; Barone, V.; Petersson, G. A.; Nakatsuji, H.; Li, X.; Caricato, M.; Crozier, A.; Marenich, P.; Bloino, J.; Janesko, B. G.; Gomperts, R.; Mennucci, B.; Hratchian, H. P.; Ortiz, J. V.; Izmaylov, A. F.; Sonnenberg, J. L.; Williams-Young, D.; Ding, F.; Lipparini, F.; Egidi, F.; Goings, J.; Peng, B.; Petrone, A.; Henderson, T.; Ranasinghe, D.; Zakrzewski, V. G.; Gao, J.; Rega, N.; Zheng, G.; Liang, W.; Hada, M.; Ehara, M.; Toyota, K.; Fukuda, R.; Hasegawa, J.; Ishida, M.; Nakajima, T.; Honda, Y.; Kitao, O.; Nakai, H.; Vreven, T.; Throssell, K.; J. A. Montgomery, J.; Peralta, J. E.; Ogliaro, F.; Bearpark, M.; Heyd, J. J.; Brothers, E.; Kudin, K. N.; Staroverov, V. N.; Keith, T.; Kobayashi, R.; Normand, J.; Raghavachari, K.; Rendell, A.; Burant, J. C.; Iyengar, S. S.; Tomasi, J.; Cossi, M.; Millam, J. M.; Klene, M.; Adamo, C.; Cammi, R.; Ochterski, J. W.; Martin, R. L.; Morokuma, K.; Farkas, O.; Foresman, J. B.; Fox, D. J., *Gaussian 09, Revision A. 02, Gaussian Inc.: Wallingford, CT* **2016**.
9. Bader, R., Atoms in Molecules: A Quantum Theory. Clarendon press: Oxford, 1990
10. Todd, A.; Keith, T. *AIMAll (Version 19.10.12), Gristmill Software*, Overland Park KS, USA, 2019.
11. Blöchl, P. E., Projector augmented-wave method. *Phys. Rev. B* **1994,** *50*, 17953–17979.
12. Kresse, G.; Marsman, M.; Furthmüller, J., Vienna Ab initio Simulation Package (VASP), the user Guide. **2010**.
13. Kresse, G.; Furthmüller, J., Efficiency of ab-initio total energy calculations for metals and semiconductors using a plane-wave basis set. *J. Comput. Mat. Sci.* **1996,** *6*, 15–50.
14. Kresse, G.; Furthmüller, J., Efficiency of ab-initio total energy calculations for metals and semiconductors using a plane-wave basis set. *Comput. Mater. Sci.* **1996,** *6* (1), 15-50, 10.1016/0927-0256(96)00008-0.
15. Kresse, G.; Hafner, J., Ab initio molecular dynamics for liquid metals. *Phys. Rev. B* **1993,** *47*, 558–561.
16. Kresse, G.; Joubert, D., From ultrasoft pseudopotentials to the projector augmented-wave method. *Phys. Rev. B* **1999,** *59*, 1758–1775.
17. Perdew, J. P.; Burke, K.; Ernzenhof, M., vasp pbe. *Phys. Rev. Lett.* **1996,** *77*, 3865–3868.
